# Cadmium toxicity induces ER stress and apoptosis via impairing energy homoeostasis in cardiomyocytes

**DOI:** 10.1042/BSR20140170

**Published:** 2015-06-22

**Authors:** Chun-yan Chen, Shao-li Zhang, Zhi-yong Liu, Yong Tian, Qian Sun

**Affiliations:** * Department of Geriatrics, The People's Hospital of Zhengzhou, Zhengzhou 450012, China; † Department of Cardiology, the First Affiliated Hospital of Xinxiang Medical University, No. 88, Rd. Jiankang, Xinxiang 453100, China; ‡ Department of internal medicine, The People's Hospital of Dezhou, Dezhou 253014, China

**Keywords:** AKT/mammalian target of rapamycin (mTOR), cardiomyocytes, cadmium toxicity, energy metabolism, endoplasmic reticulum (ER) stress

## Abstract

Cadmium, a highly toxic environmental pollutant, is reported to induce toxicity and apoptosis in multiple organs and cells, all possibly contributing to apoptosis in certain pathophysiologic situations. Previous studies have described that cadmium toxicity induces biochemical and physiological changes in the heart and finally leads to cardiac dysfunctions, such as decreasing contractile tension, rate of tension development, heart rate, coronary flow rate and atrioventricular node conductivity. Although many progresses have been made, the mechanism responsible for cadmium-induced cellular alternations and cardiac toxicity is still not fully understood. In the present study, we demonstrated that cadmium toxicity induced dramatic endoplasmic reticulum (ER) stress and impaired energy homoeostasis in cultured cardiomyocytes. Moreover, cadmium toxicity may inhibit protein kinase B (AKT)/mTOR (mammalian target of rapamycin) pathway to reduce energy productions, by either disrupting the glucose metabolism or inhibiting mitochondrial respiratory gene expressions. Our work will help to reveal a novel mechanism to clarify the role of cadmium toxicity to cardiomyocytes and provide new possibilities for the treatment of cardiovascular diseases related to cadmium toxicity.

## Abstract (precis)

Cadmium, a highly toxic environmental pollutant, is reported to induce toxicity and apoptosis in multiple organs and cells. The present findings showed that cadmium toxicity induces cell stress and promotes apoptosis in cardiomyocytes in a metabolic manner, by either disrupting the glucose metabolism or inhibiting mitochondrial respiratory gene expressions through AKT/mTOR pathway.

## INTRODUCTION

Cadmium is an environmental pollutant and a highly toxic metal ion, accumulated largely in the liver and kidney [1,2]. Chronic exposure of humans to a cadmium-contaminated environment or food chain may be implicated in some human disorders, such as obstructive pulmonary disease [3], renal tubular dysfunction [4], hypertension and even arteriosclerotic heart diseases [5–7]. Indeed, studies in animals or cultured cells have implicated cadmium in aetiology and pathogenesis of hypertension and toxicity [8–10]. Under acute or chronic cadmium exposure conditions, cardiac toxicity occurs earlier than hepatic or renal toxicity. Therefore, cadmium intoxication has been considered and studied as a possible aetiological factor in cardiovascular diseases.

Heart muscle cells have a high and varying requirement for metabolic energy, the demand increased as contraction stimulation [11]. To meet the energy demand in the heart, energy production of the heart is primarily dependent on mitochondria for ATP production by glucose oxidation [12]. When transported into the heart cells, glucose is oxidized by glycolysis in cytosol and tricarboxylic acid (TCA) cycle in mitochondria, to form ATP from ADP and inorganic phosphate (P_i_). Although many metabolites involved in cardiac energy metabolism are held constant in homoeostasis over the range of normal physiological condition, environmental toxicity and cell stress may imbalance the metabolic homoeostasis and disrupt the energy production in the heart. Once the energy production is blocked (e.g., by ischaemia or hypoxia), metabolic homoeostasis of the heart may be impaired, leading to cardiac cell death and various cardiovascular diseases [13].

It has been long appreciated that cadmium toxicity induces biochemical and physiological changes in the heart [14,15]. For example, cadmium perfusion significantly decreased contractile tension, rate of tension development, heart rate, coronary flow rate and atrioventricular node conductivity [16]. Moreover, cadmium acted solely as an inhibitor of rat heart pyruvate-malate supported mitochondrial respiration [17]. And cadmium treatment of rats decreased activities of the selenoenzyme, glutathione peroxidase and copper-containing enzyme superoxide dismutase, together with a rise in thiobarbiturate-reactive substances in the heart [9]. Thus, cadmium toxicity is associated with a variety of cellular metabolic, homoeostatic and repair mechanisms, such as increased production of reactive oxygen species (ROS) and activation of apoptosis. Whereas biochemical and physiological changes in the heart as a result of cadmium exposure have been reported, molecular elements involved in cadmium toxicity in cardiovascular diseases are not fully characterized. Understanding the mechanism of cadmium-induced cell alternations in cardiomyocytes, therefore is an essential step toward delineating the mechanism of cadmium-induced toxicity.

To determine the mechanism of how cadmium toxicity affects cardiac cell functions, we investigated the cell alternations after gradient concentrations of cadmium treatment. Our results showed that cadmium treatment-induced dramatic endoplasmic reticulum (ER) stress in cardiomyocytes and thus led to apoptosis. Since cardiomyocytes were highly dependent on energy productions, cadmium toxicity disrupted glucose consumptions and utilizations through inhibiting AKT/mTOR (mammalian target of rapamycin) pathway. Moreover, cadmium toxicity also impaired cardiac mitochondrial respiratory gene expression, promoting energy reductions. Taken together, our work reveals cadmium-induced metabolic alternations in cardiomyocytes and will help to understand the mechanism of cadmium cardiac toxicity, indicating that energy metabolism serves as a target for cadmium intoxication.

## MATERIAL AND METHODS

### Chemicals and reagents

The toxic cadmium chloride (CdCl_2_) was obtained from Sigma Chemical Co.. Dulbecco's Modified Essential Medium (DMEM) and FBS were purchased from GIBCO Invitrogen. The antibodies of anti-GRP78 (glucose-regulated protein 78)/BiP (binding immunoglobulin protein), anti-p-eIF2α (phosphorylates eukaryotic initiation factor 2α), anti-eIF2α, anti-pro- and cleaved-caspase 3, anti-pAKT, anti-AKT, anti-p-p70S6K and anti-p70S6K were purchased from Cell Signaling Technology. Whereas CCAAT/enhancer-binding protein homology protein (CHOP) and actin antibodies were from Millipore. The Hoechst kit for cell death detection was from Beyotime Biotechnology. Metabolic kits for glucose, pyruvate and ATP examinations were from Biovision. Other chemicals were purchased from Sigma unless specified, which were of the highest purity available.

### Cell culture and cadmium treatment

For *in vitro* experiment, primary cardiomyocytes were prepared from the cardiac ventricles of 17–19-day (gestational age) Sprague–Dawley rats according to described protocols [18]. The cardiomyocytes were grown in six- or 12-well plates at 1.0 × 10^6^ cells/ml in DMEM with 10% FBS plus antibiotics and insulin for in 5% CO_2_ at 37°C. For the cadmium treatment, the final low and high concentrations (1 and 100 μM) of cadmium were applied to these cells, incubated for 4 or 12 h. Equivalent DMSO was used as internal controls. After culturing and cadmium treatment, cells were harvested for subsequent examinations.

### Quantitative real-time PCR

Total RNA was extracted from tissues using TRizol reagent (Invitrogen). RNA was subjected to reverse transcription with reverse transcriptase as per manufacturer's instructions (Fermentas). Quantitative real-time PCR was performed using the Bio-Rad iQ5 system and the relative gene expression was normalized to internal control using β-actin. Primer sequences for SYBR Green probes of target genes are shown as Table 1. The amplification efficiency and specificity were confirmed before applying them to the assay.

**Table 1:** Primer sequences for SYBR Green probes of target genes

Name	Primer sequence (5′→3′)
*Grp78-F*	CGCTTCGAATCGGCGGTACCCAG
*Grp78*-R	TCCTTCTTGTCCTCCTCCTA AGCTTCGCG
*Atf4*-F	GTTGGTCAGTGCCTCAGACA
*Atf4*-R	CATTCGAAACAGAGCATCGA
*Atf6*-F	AACAAGACCACAAGACCAA
*Atf6*-R	AGGAGGAACTGACGAACT
*Pgc1-α*-F	CTCCCTGTGGATGAAGACGG
*Pgc1-α*-R	GCAAATC ACAATCACAGGAT
*Nrf1*-F	CTTTCTGCCTCAGGTGGAACA
*Nrf1*-R	GA TGCTTGCGTCGTCTGGAT
*Actin*-F	TGGCATTGTGATGGACTCCG
*Actin*-R	TTAATGTCACGCACGATTTCCC

### Cell lysates preparation and western blots

Western blot was performed as standard procedures. In brief, cardiomyocyte samples were lysed in lysis buffer (PBS with 1% Triton X-100 and protease inhibitors) and insoluble material was removed by centrifugation at 12000 ***g*** for 20 min at 4°C. Final protein concentrations were determined using the Bradford protein assay (Bio-Rad). Electrophoresis was performed using SDS/PAGE and blots were transferred to nitrocellulose membranes. Membranes were incubated with appropriate primary antibodies and secondary antibodies. Membranes were then visualized using an enhanced chemiluminescent technique. Resulting films were scanned and optical densities were quantified using National Institutes of Health software ImageJ, which is available from http://rsbweb.nih.gov.

### Detection of cell apoptosis and viability

For the preparation of cell death detection by Hoechst staining, cardiomyocytes were plated with 1.0 × 10^5^ cells/ml in six-well plates. After cadmium treatments, these cells were directly stained with Hoechst kit from Beyotime. The cell counting was carried out through the use of ImageJ for quantifications. For the preparation of cell viability by MTT assay, cells were treated in 96-well plate, the viable cells were stained by adding 20 μl of 5 mg/ml MTT solution per 100 μl of growth medium. After incubating for 4 h at 37°C, the media were removed and 150 μl of DMSO was added to dissolve the formazan. The absorbance of each well was measured by microplate reader and viable cells are presented as a percent of the control.

### Metabolic examination

All the metabolic examinations, including glucose consumptions, pyruvate and ATP productions, were all performed according to manufacturer instructions of Biovision. Briefly, a total of 1 × 10^6^ cells per well were seeded in six-well plates, with or without cadmium treatments. Then cells were washed, harvested and homogenized in assay buffer to be prepared to samples and media were collected for glucose consumption examinations. Samples were mixed with respective reaction buffers and read at fluorescence at *E*_x_/*E*_m_=535/590 nm in a microplate reader to measure products concentrations. All the final results were normalized to cell numbers for quantifications.

### Statistical analysis

All statistical analysis was performed by ImageJ software. Quantitative data were showed in x^−^ ± s using ANOVA tests for comparisons. The value 0.05 (*), 0.01 (**) and 0.001 (***) was assumed as the level of significance for the statistic tests.

## RESULTS

### Cadmium toxicity induces ER stress in cardiomyocytes

Cadmium is reported to produce toxicity at doses and exposure conditions. However, the toxic effects of cadmium on cardiomyocytes are not fully elucidated. To characterize the toxic effects of cadmium on cardiomyocytes, we examined cell alternations induced by cadmium. It has been known that heavy metal toxic could impair ER homoeostasis and induce unfolded protein response (UPR) and ER stress [19,20]. To study whether cadmium toxicity would disrupt ER homoeostasis in cardiomyocytes, we applied cultured cardiomyocytes incubated with gradient concentrations of cadmium for 4–12 h. The real-time PCR results showed that mRNA levels of *Grp78*, *Atf4* and *Atf6*, which were canonical markers of UPR and ER stress, increased dramatically by cadmium treatment in a dose- and time-dependent manner (Figures 1A–1C). To further confirm the increased ER stress by cadmium treatment in cardiomyocytes, we examined the protein levels of UPR markers, GRP78/BiP and p-eIF2α. It is showed that protein levels of these UPR markers were also increased by cadmium treatment (Figures 1D and 1E). Notably, higher cadmium concentrations and longer cadmium-treated times would enhance the ER toxicity of cadmium in cardiomyocytes. Collectively, these results suggest that cadmium treatment may impair ER homoeostasis and promote ER stress in cardiomyocytes, which may disrupt normal metabolism and lead to cardiac cell death.

**Figure 1:**
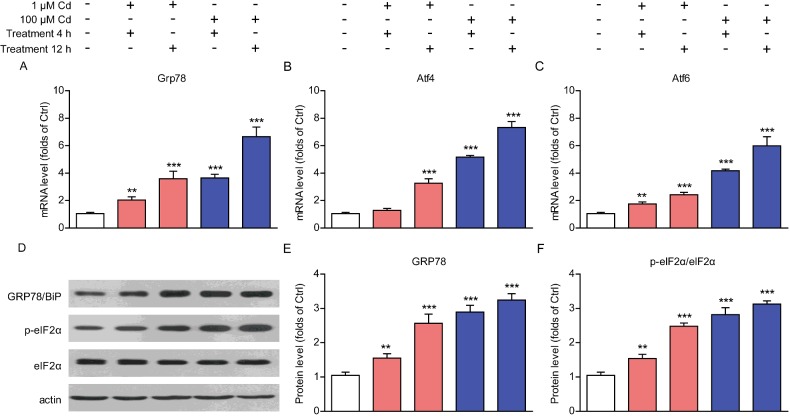
Cadmium toxicity induces ER stress in cardiomyocytes (**A**–**C**) Real-time PCR results showing the increasing of UPR genes *Grp78* (**A**), *Atf4* (**B**) and *Atf6* (**C**) in cardiomyocytes treated by cadmium (1 or 100 μM for 4–12 h). (**D** and **E**) Western blots and histograms showing the increasing of GRP78/BiP and p-eIF2α protein levels in cardiomyocytes treated by cadmium (1 or 100 μM for 4–12 h). Results are averages of three independent experiments. Data represent mean ± S.E.M. **P<0.01 and ***P<0.001.

### Cadmium toxicity induces cell apoptosis in cardiomyocytes

It is reported that ER stress may lead to cell apoptosis in several types of primary cells and animal models [21]. Since cadmium treatment may induce ER stress in cardiomyocytes, we would like to study whether the subsequent cell apoptosis occurred. To examine the effect of cadmium on cell apoptosis in cardiomyocytes, we carried out Hoechst staining and MTT assay to visualize the cell status. The results showed that by cadmium treatment, dramatic cell apoptosis were found in cardiomyocytes. Similarly, higher cadmium concentrations and longer cadmium-treated times would enhance the apoptosis in cardiomyocytes, for example, cadmium of 100 μM for 12 h induced nearly 41.7% apoptotic cardiomyocytes. Consistently, the MTT results confirmed that cell viability of cardiomyocytes was reduced as cadmium treatment (Figure 2A). This is consistent with the alternations of UPR in cadmium-treated cardiomyocytes, suggesting that ER stress may contribute to the cardiomyocyte apoptosis. To confirm these findings, we carried out western blots to analyse the apoptotic protein levels in cadmium-treated cardiomyocytes. Indeed, we found that the protein levels of CHOP and cleaved-caspase 3, well-known apoptotic proteins, were increased by cadmium treatment in a dose- and time-dependent manner (Figures 2B–C). Thus, our results strongly indicate that cadmium toxicity may remarkably lead to apoptosis in cardiomyocytes.

**Figure 2:**
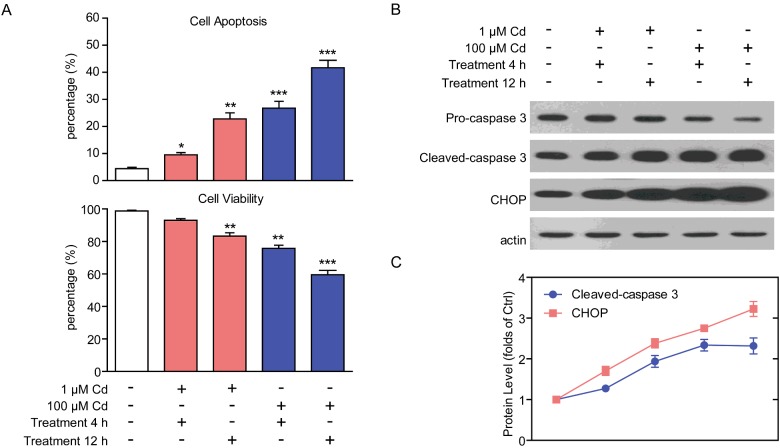
Cadmium toxicity induces cell apoptosis in cardiomyocytes (**A**) Histograms showing the quantification of the cell apoptosis (%) and viability (%) in cardiomyocytes by cadmium (1 or 100 μM for 4–12 h) treatment. (**B** and **C**) Western blots and quantifications showing the protein levels of cleaved-caspase 3 and CHOP increased by cadmium (1 or 100 μM for 4–12 h) treatment in cardiomyocytes. Results are averages of four independent experiments. Data represent mean ± S.E.M. *P<0.05, **P<0.01 and ***P<0.001.

### Cadmium toxicity reduces energy production in cardiomyocytes

We have proved that cadmium treatment may induce ER stress in cardiomyocytes; whereas ER stress could also affect cellular metabolism and energy production [22]. Thus, we proposed that cadmium toxicity may impair the energy production in cardiomyocytes. To examine the energy status, we applied luminescence assays to determine the ATP levels by cadmium treatment in cardiomyocytes. The results showed that cadmium treatment indeed decreased the ATP levels in cardiomyocytes and the ATP level may reduce even by 30.3% after 100 μM for 12 h of cadmium treatment (Figure 3). Therefore, these results verify that cadmium toxicity may impair energy homoeostasis in cardiomyocytes.

**Figure 3:**
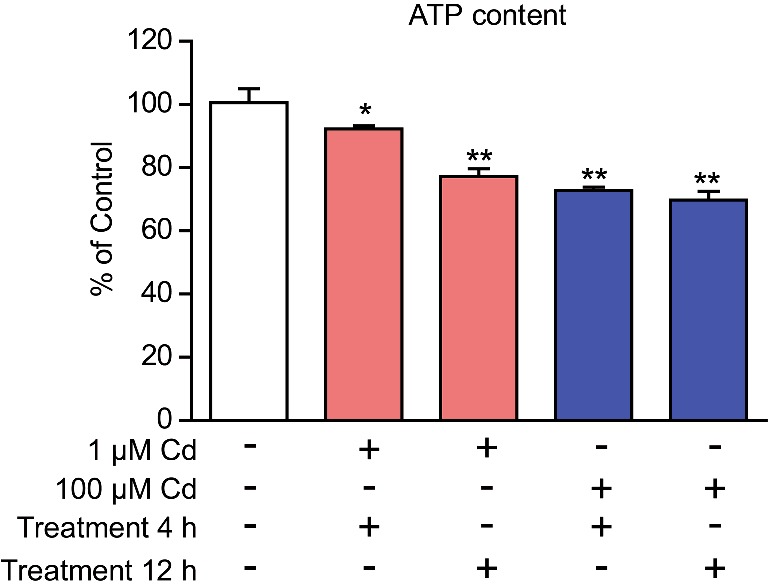
Cadmium toxicity reduces energy production in cardiomyocytes Histograms showing that ATP levels were reduced by cadmium (1 or 100 μM for 4–12 h) treatment in cardiomyocytes. Results are averages of four independent experiments. Data represent mean ± S.E.M. *P<0.05 and **P<0.01.

### Cadmium toxicity impairs glucose metabolism in cardiomyocytes

In mammalian cells, most energy productions are derived from glucose oxidation, including glycolysis and TCA cycle. Since cadmium toxicity decreased ATP productions in cardiomyocytes, we would like to study whether glucose metabolism is affected by cadmium toxicity. Using biochemical assays, we quantified the glucose consumptions and pyruvate productions (indicator of glycolysis) in cardiomyocytes by cadmium treatment. Results showed that consistent reduction in glucose consumptions and pyruvate productions were found in cadmium-treated cardiomyocytes. Cadmium treatment of 100 μM for 12 h terribly decreased glucose consumptions and pyruvate productions to 56.8% and 50.6% of controls (Figures 4A and 4B). Cardiomyocytes are highly dependent of glucose to serve as energy supply, thus the reduced glucose metabolism may be responsible for the reduced ATP productions in cadmium-treated cardiomyocytes.

**Figure 4:**
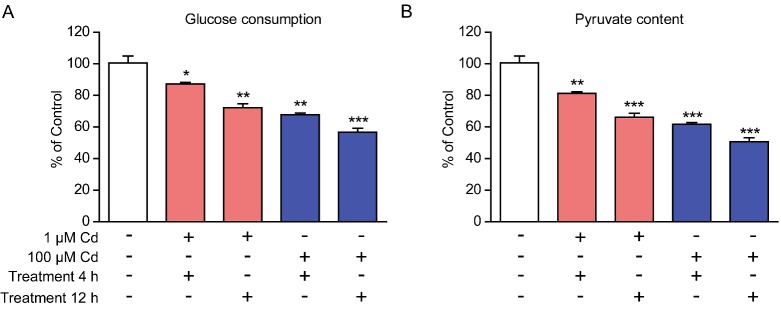
Cadmium toxicity impairs glucose metabolism in cardiomyocytes Biochemical results showing that glucose consumptions (**A**) and pyruvate productions (**B**) were reduced by cadmium (1 or 100 μM for 4–12 h) treatment in cardiomyocytes. Results are averages of four independent experiments. Data represent mean ± S.E.M. *P<0.05, **P<0.01 and ***P<0.001.

### Cadmium toxicity down-regulates AKT/mTOR signalling

To investigate the mechanism of how cadmium toxicity impairs glucose metabolism in cardiomyocytes, we next examined the cellular pathways modulating glucose metabolism. AKT/mTOR pathway is a central regulator of cellular metabolism in various cultured cells and animal models [23,24]. For example, AKT activity may promote glucose transporter surface expressions and increase glucose uptake [25]. Whereas mTOR controls glycolysis via modulating glycolytic gene transcriptions or enzyme activities [26]. Therefore, we assumed that cadmium toxicity may impair glucose metabolism via inhibiting AKT/mTOR pathway. The biochemical results showed that AKT activity (indicated by pAKT/AKT) and mTOR activity (indicated by p-p70S6K/p70S6K) were both gradiently decreased by cadmium treatment (Figures 5A and 5B). Of note, the mTOR activity seemed to be more sensitive to cadmium toxicity, indicated that mTOR may be a toxicant target of cadmium in cardiomyocytes. Taken together, all these data indicates that cadmium toxicity may inhibit AKT/mTOR pathway, which may contribute to the decreased glucose oxidations and energy productions in cardiomyocytes.

**Figure 5:**
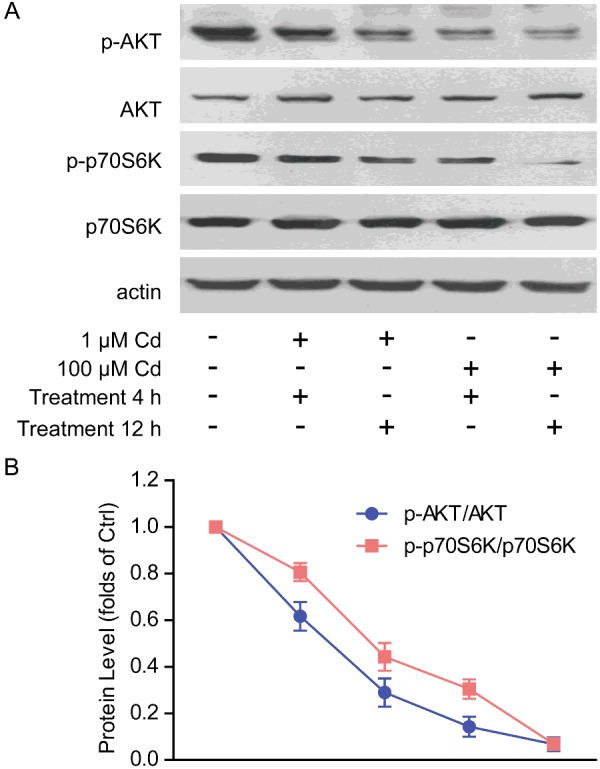
Cadmium toxicity down-regulates AKT/mTOR signalling Western blots (**A**) and quantifications (**B**) showing the reduction in AKT activity (indicated by p-AKT/AKT) and mTOR activity (indicated by p-p70S6K/p70S6K) by cadmium (1 or 100 μM for 4–12 h) treatment in cardiomyocytes. Results are averages of three independent experiments. Data represent mean ± S.E.M.

### Cadmium toxicity affects mitochondrial gene expression in cardiomyocytes

Cadmium toxicity has been showed to impair energy productions in cardiomyocytes, as well as glucose oxidations. It has been reported that cadmium acted solely as an inhibitor of rat heart pyruvate–malate-supported mitochondrial respiration [17]. Considering that glucose oxidation primarily occurs in mitochondria, we would like to confirm whether mitochondria respiratory gene expression is altered by cadmium toxicity in cardiomyocytes. Real-time RCR results showed that cadmium toxicity indeed inhibited gene transcriptions of peroxisome proliferator-activated receptor-γ coactivator-1α (*Pgc1-α*) and nuclear respiratory factor 1 (*Nrf1*), which were master regulators of various mitochondrial respiratory gene expressions. Of note, the reduced gene transcriptions of *Pgc1-α* and *Nrf1* were enhanced by higher cadmium concentrations or longer cadmium-treated times (Figures 6A and 6B). Therefore, cadmium toxicity may affect mitochondrial gene expression via inhibiting *Pgc1-α* and *Nrf1*, which may contribute to the energy reduction in cadmium-treated cardiomyocytes.

**Figure 6:**
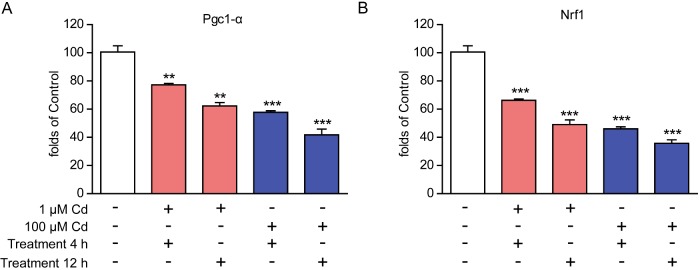
Cadmium toxicity affects mitochondrial gene expression in cardiomyocytes Real-time PCR results showing the reduction in mitochondrial regulatory gene *Pgc-1α* and *Nrf1* by cadmium (1 or 100 μM for 4–12 h) treatment in cardiomyocytes. Results are averages of four independent experiments. Data represent mean ± S.E.M. **P<0.01 and ***P<0.001.

## DISCUSSION

Cadmium toxicity has long been appreciated as an inducer of various cardiovascular diseases. However, molecular mechanism and cardiac cell alternations by cadmium treatment are still not well characterized. In the present study, we reveal a novel mechanism to clarify the role of cadmium toxicity to cardiomyocytes. We demonstrate that cadmium treatment induced dramatic ER stress in cardiomyocytes and thus led to cardiac cell death. Since cardiomyocytes were highly dependent on energy productions, cadmium toxicity disrupted glucose consumptions and pyruvate productions through inhibiting AKT/mTOR pathway. Moreover, cadmium toxicity also impaired cardiac mitochondrial respiratory gene expression, which enhanced energy reductions (Figure 7). Our work will help to provide new possibilities for treatment of cardiovascular diseases related to cadmium toxicity.

**Figure 7:**
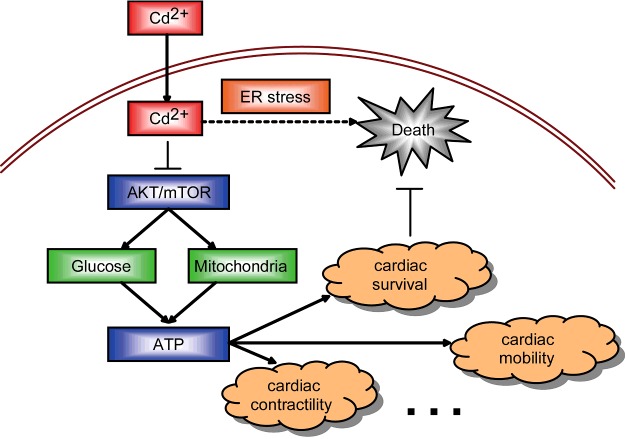
Model Schematic representation highlighting the toxicity of cadmium in cardiomyocytes. The cardiotoxicity of cadmium induces ER stress and promotes apoptosis in cardiomyocytes in a metabolic manner, by either disrupting the glucose metabolism or inhibiting mitochondrial respiratory gene expressions through AKT/mTOR pathway.

Cadmium is a toxic heavy metal, responsible for obstructive airway diseases, emphysema, end-stage renal failures, diabetic and renal complications, deregulated blood pressure, bone disorders and various cardiovascular diseases [2]. The cardiomyocytes displayed toxicity at a cadmium concentration that was 100-fold lower than that reported to be toxic for liver and kidney cells [15]. The extreme sensitivity of cardiomyocytes to cadmium may be related to the abundance of calcium channels in these cells, which allow efficient uptake of cadmium whereas blocking calcium uptake [15]. In the present study, we described that cadmium toxicity in cardiomyocytes may be mediated in a metabolic manner. The reduced glucose metabolism and energy production impairs the normal structures and functions of cardiomyocytes, inducing cellular stress and finally leading to cell apoptosis. Our work is consistent with previous studies showing that cadmium is highly toxic metal ion in cardiovascular system and offers a novel insight that cadmium toxicity may target at energy metabolism besides activation apoptotic pathways [27] and increasing of ROS productions [28]. Therefore, improvement of energy metabolism may be necessary to therapy of cadmium intoxication diseases.

The heart is nearly unique in the body in that it has a constant workload well beyond the normal maintenance of cellular integrity [29]. In addition, the heart of a large animal such as man also has the capacity to increase its workload by nearly 10-fold for considerable amounts of time [12]. A high steady state and peak work demand require the heart to use the space- and weight-efficient mitochondria to provide an adequate energy production supporting these activities [30]. Once the energy production is blocked or disrupted, there may be terrible cardiac cell death and subsequent cardiovascular diseases. The toxicity of cadmium has been reported to be highly correlated with the cardiovascular diseases, but the mechanisms and cellular alternations are not fully understood.

In the present study, our results suggested that cadmium toxicity may induce ER stress and reduce energy production, by either disrupting glucose metabolism or inhibiting mitochondrial respiratory gene expressions. These two aspects of cadmium toxicity on cardiomyocytes would terribly impair the energy homoeostasis. Therefore, it's of possibility that drugs to improve glucose utilization and mitochondrial functions may be beneficial to the therapy of cadmium poisoning. Further study will be needed to gain additional details about targets of intracellular cadmium inducing ER stress and reducing energy productions, setting stages to the clinical treatment of related cardiovascular diseases.

In conclusion, the results of the present study support the notion that toxicity of cadmium induces cell stress and promotes apoptosis in cardiomyocytes in a metabolic manner, by either disrupting the glucose metabolism or inhibiting mitochondrial respiratory gene expressions through AKT/mTOR pathway. All these results will help to understand the mechanism of cadmium toxicity, indicating that energy metabolism serves as a target for cadmium intoxication.

## Abbreviations

DMEM: Dulbecco’s Modified Essential Medium
ER: endoplasmic reticulum
mTOR: mammalian target of rapamycin
ROS: reactive oxygen species
TCA: tricarboxylic acid
UPR: unfolded protein response
